# A Retrospective Study on Mucous Stool Excretion in Giant Pandas: Statistical Analysis Based on CCRCGP Data

**DOI:** 10.3390/ani16193047

**Published:** 2026-09-28

**Authors:** Pingfeng Wang, Xiaomei Zheng, Minfu Feng, Denghe Li, Shuhong Xie, Li Dong, Xinyan Wu, Shanshan Ling

**Affiliations:** 1 China Conservation and Research Centre for the Giant Panda, Chengdu 610081, China; 2 Key Laboratory of SFGA on the Giant Panda, Chengdu 610081, China; 3 Yingjing County Daxiangling Nature Reserve Management and Protection Center, Ya’an 625200, China; 4 College of Food Science and Nutritional Engineering, China Agricultural University, Beijing 100083, China

**Keywords:** *Ailuropoda melanoleuca*, mucous stool excretion, monthly variation, captive management

## Abstract

This retrospective multicenter study integrated individual- and event-level records from 190 captive giant pandas managed at four study sites of the China Conservation and Research Center for the Giant Panda (CCRCGP) during 2024 to characterize patterns of mucous stool excretion and associated phenotypic variation. Logistic and negative binomial regression models were used to evaluate individual-level associations with event occurrence and annual event frequency, while generalized estimating equations (GEEs) were applied to monthly and event-level analyses to account for repeated observations within pandas. Overall, 132 of 190 pandas (69.5%, 95% CI, 62.6–75.6%) experienced at least one mucous stool event, with 674 events recorded during the study year. Monthly event frequency differed significantly overall (Poisson GEE, *p* = 0.0015), with the largest observed event count occurring in July, although no individual month-versus-January comparison remained significant after Holm correction. The monthly probability of an individual panda experiencing at least one event did not differ significantly (*p* = 0.295). Mucous stool weight showed no significant monthly variation (*p* = 0.453) but differed markedly by physical phenotype, compared with jelly-like stools, lard-like and mixed jelly/lard stools were 1.318- and 2.133-fold heavier, respectively. An exploratory inverse association between body weight and event occurrence persisted after adjustment for age and sex (adjusted OR per kg = 0.961, 95% CI, 0.930–0.992, *p* = 0.015). Recurrently affected individuals contributed disproportionately to the annual event burden, but did not exclusively account for the elevated July count. These findings indicate that within-year variation primarily reflected changes in event burden rather than the proportion of affected pandas, while visible phenotype was strongly associated with the amount of mucous material excreted. The disproportionate contribution of recurrently affected individuals highlights the value of targeted longitudinal monitoring in veterinary and husbandry management, while multi-year prospective surveillance is needed to confirm reproducible temporal patterns and clarify underlying mechanisms.

## Simple Summary

Mucous stool excretion is commonly observed in captive giant pandas, but its occurrence, recurrence, and visible characteristics are not fully understood. This study reviewed routine health and husbandry records from 190 captive giant pandas during 2024 to describe how often mucous stool events occurred, whether they varied across the year, and whether stool appearance was related to the amount of material excreted. A total of 674 mucous stool events were recorded, involving 132 pandas. Event numbers varied across months, with the largest number observed in July, although the proportion of pandas affected each month did not change significantly. A small group of repeatedly affected pandas contributed a substantial share of all events. Mucous stool weight did not differ clearly across months, but mixed jelly-like and lard-like stools were heavier than jelly-like stools. These findings suggest that repeated episodes in susceptible individuals and visible stool characteristics are important for understanding this phenomenon. The results may help improve routine monitoring, record keeping, and individualized health management of captive giant pandas, supporting better animal welfare and conservation practice.

## 1. Introduction

The giant panda (*Ailuropoda melanoleuca*) has a highly specialized bamboo-based diet despite having a relatively simple gastrointestinal tract [1]. Intestinal mucus helps protect the gastrointestinal mucosa from mechanical and microbial challenges [2]. In captive giant pandas, visible masses of mucus are sometimes passed as mucous stools (Figure 1). These episodes are often accompanied by reduced appetite, decreased activity, abdominal discomfort, and other temporary signs of gastrointestinal disturbance [3,4]. As mucous stool episodes can last from one to several days, they may affect both gastrointestinal health and the daily management and welfare of captive pandas.

**Figure 1:**
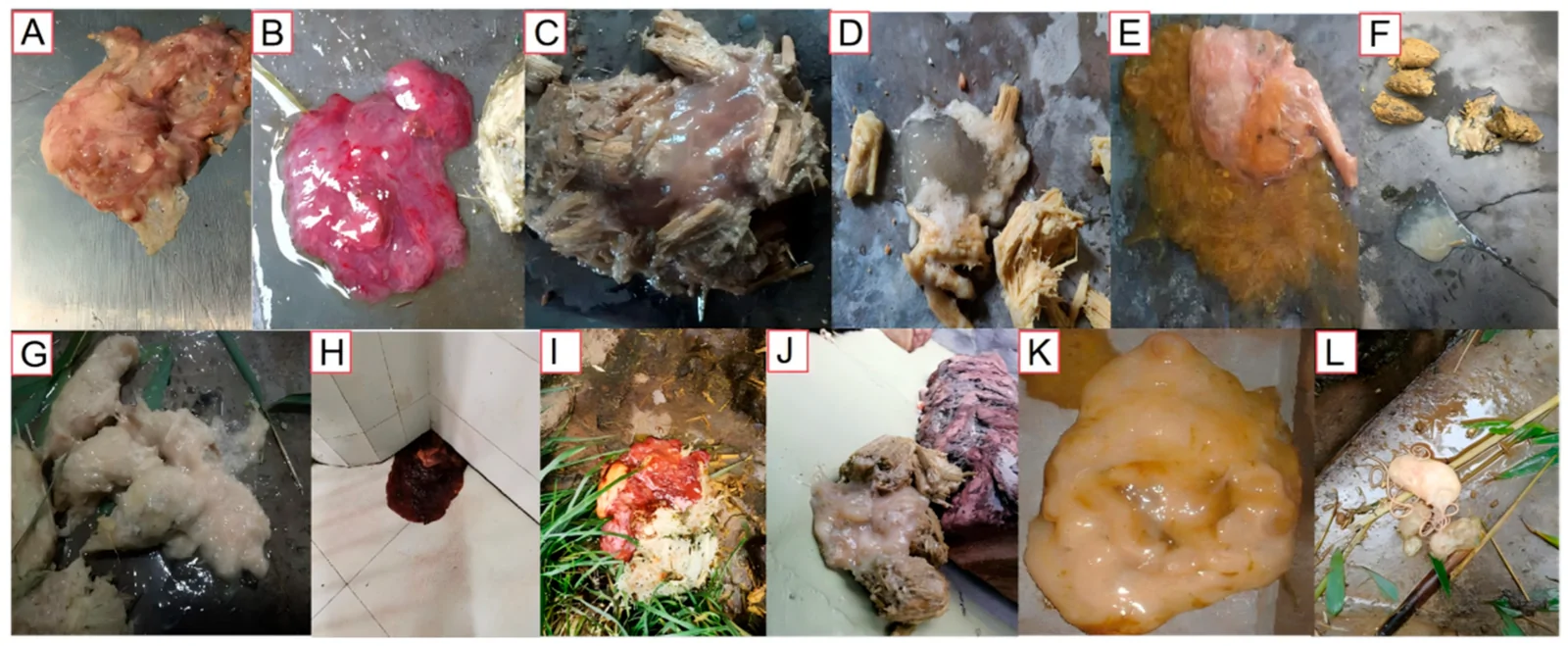
The common mucus characteristics of the giant panda (**A**,**B**) pink, lard-like mucus; (**C**,**D**) semi-transparent, jelly-like mucus; (**E**) light yellow, lard-like jelly mucus; (**F**) transparent, jelly-like mucus; (**G**) milky white, lard-like mucus; (**H**) pink, lard-like mucus with blood; (**I**) light yellow, lard-like mucus with blood; (**J**) milky white, lard-like mucus; (**K**) light yellow, lard-like mucus; and (**L**) milky white, lard-like mucus with roundworms. The standardized physical phenotype and color categories illustrated in these panels, where applicable, correspond to the categories summarized quantitatively in Table 1.

**Table 1:** Descriptive statistics on basic characteristics of giant pandas.

Characteristic	Overall N = 190	Female N = 103	Male N = 86
Age	14 (6)	14 (6)	13 (6)
Body weight (kg)	117 (13)	112 (11.8)	121 (11.8)
Mucous stool weight (g) *	69.1 (55.1)	64.3 (48.7)	75.2 (61.6)
Physical phenotype * (*n* = 672)			
Jelly-like	330 (49.1%)	178 (47.7%)	152 (50.8%)
Mixed jelly/lard	53 (7.9%)	26 (7.0%)	27 (9.0%)
Lard	289 (43.0%)	169 (45.3%)	120 (40.1%)
Color (*n* = 674)			
Brown	8 (1.2%)	4 (1.1%)	4 (1.3%)
Chartreuse	4 (0.6%)	3 (0.8%)	1 (0.3%)
Milky white	64 (9.5%)	33 (8.8%)	31 (10.3%)
Pale yellow	63 (9.3%)	34 (9.1%)	29 (9.7%)
Pink	44 (6.5%)	22 (5.9%)	22 (7.3%)
Transparent	5 (0.7%)	5 (1.3%)	0 (0%)
Not recorded	486 (72.1%)	273 (73.0%)	213 (71.0%)

Values are mean (SD) or *n* (%). Percentages for physical phenotype and color were calculated among events with non-missing data for the corresponding variable. The denominators were 672 events for physical phenotype (female *n* = 373; male *n* = 299) and 188 events for color (female *n* = 101; male *n* = 87). Missing-data frequencies are reported separately in Supplementary Table S1. * For mucous stool weight and physical phenotype, summary statistics were calculated using events with available data for the corresponding variable (mucous stool weight, *n* = 670; physical phenotype, *n* = 672).

Previous studies have suggested that feeding and dietary factors may contribute to mucous stool excretion. Reduced bamboo intake, changes in bamboo composition, and transitions between plant parts have all been proposed as potential contributors [5,6,7,8]. Dietary shifts can also alter the giant panda gut microbiome and have been linked to dysbiosis and mucous stools. A 15-year prospective study of 18 giant pandas further showed that mucous stool occurrence varied by month and was associated with age and daily fecal output, but not sex [4]. These findings indicate that mucous stool excretion may reflect interactions among individual susceptibility, diet, seasonal husbandry conditions, and gastrointestinal physiology.

Important gaps remain. Previous studies often involved small populations, single institutions, or limited event-level phenotyping. Although long-term prospective monitoring provides detailed longitudinal information, extending such intensive daily follow-up to a much larger multicenter population is challenging. The present retrospective design therefore represents a complementary trade-off: it enables broader population coverage across four study sites and greater statistical power for evaluating individual- and event-level patterns, but provides a shorter observation window and less granular event-level information than prospective daily monitoring. The amount of mucous material excreted and its relationship with visible phenotype have also rarely been evaluated at scale, and multicenter datasets require methods that account for repeated events within the same individual.

Based on previous observations and the biological heterogeneity of mucous stool events, our primary hypotheses were that (1) mucous stool event frequency would vary across the study year, without necessarily being accompanied by parallel variation in the proportion of pandas affected, and (2) the amount of mucous material excreted would differ according to visible physical phenotype. Associations of age and sex with event occurrence and annual event frequency were also evaluated as prespecified individual-level analyses. Body weight was examined as an exploratory factor because of incomplete and non-standardized measurements, whereas color was retained for descriptive purposes only because of substantial missing event-level documentation. Accordingly, the objectives were to (1) describe the basic characteristics and phenotypic spectrum of mucous stool events, (2) evaluate associations of age and sex with whether mucous stool excretion occurred and with annual event frequency, (3) characterize within-year variation in event frequency and the proportion of affected pandas, and (4) determine whether mucous stool weight varied by month or physical phenotype.

## 2. Materials and Methods

### 2.1. Subjects and Management

This retrospective multicenter study used health records from four study sites of CCRCGP: the Ya’an Base, Wolong Shenshuping Base, Dujiangyan Base, and Wolong Hetaoping Base (Figure 2). Giant pandas were not permanently assigned to a single study site and could be transferred among CCRCGP bases during routine management. Therefore, some individuals may have contributed records from more than one site during 2024, and a mutually exclusive individual-level site classification was not applicable. Complete time-resolved residence histories were not consistently available for retrospective reconstruction. Records from the four sites were pooled for the present analysis under the standardized husbandry and management framework described below. All four CCRCGP bases followed the same institutional husbandry and feeding framework under the standardized “Operating Procedures for Giant Panda Breeding and Management.” However, environmental and climatic exposures were site-specific, and some feeding-related factors, including bamboo species and plant parts provided, could vary according to season and local availability. Because the study relied on retrospective routine records, complete uniformity in mucous-stool weighing, phenotype classification, and event-recording procedures across the four bases could not be verified. Event-level terminology and categorical variables were therefore harmonized during data cleaning prior to analysis. Residual site-level heterogeneity was considered when interpreting the findings. After data cleaning and reconciliation of individual identifiers, the final analytic dataset comprised 190 giant pandas. Records covered the 2024 observation period. Age was calculated using 1 July 2024 as a common reference date for all pandas with an available date of birth. All pandas included in the analytic population were under observation throughout the 2024 study period, with no mid-year entry or exit due to birth, death, or transfer into or out of the study population.

All 190 giant pandas included in the study were managed during the 2024 observation period under the standardized CCRCGP “Operating Procedures for Giant Panda Breeding and Management,” and the husbandry and feeding practices described below therefore represent the routine management framework applied to the study cohort. Within this standardized framework, some feeding-related details, particularly bamboo species, plant parts, and amounts provided, could vary according to season, local availability, age, reproductive status, and individual condition.

The daily feeding plan includes the following: panda cake feed—feed 0.6 to 2.0 kg of panda cake feed daily; auxiliary fruits and vegetables—feed 0.4 to 0.8 kg of carrots and approximately 0.2–0.5 kg of apples daily; bamboo and bamboo shoots—20–40 kg of fresh bamboo are provided daily, and the bamboo species are changed according to the season. The feeding amount of bamboo shoots is adjusted according to the season, age of giant pandas, reproductive status (such as litter), and other actual conditions. The parts where giant pandas feed on bamboo also vary depending on the bamboo species. Living environment: Each giant panda has an independent inner house and outer playground, and the living area is equipped with abundant facilities such as shelters, caves, and water pools to promote its natural behavior.

### 2.2. Data Collection and Variable Definitions

Individual-level variables included name, sex, studbook number, date of birth, and body weight when available. Body-weight measurements were derived from routine management records and were not obtained at a standardized study-specific time point relative to the 2024 observation period or to individual mucous stool events. Exact weighing dates were not consistently available and therefore could not be reliably reconstructed. Event-level variables included date of mucous stool excretion, mucous stool weight (g), visible physical phenotype and color. Each row in the event-level analytic dataset represented one mucous stool event. Before statistical analysis, source records were harmonized and reconciled at both the individual and event levels. Individual identity was cross-checked using studbook number, animal name, and recorded date of birth so that repeated records from the same panda were linked consistently. Date-of-birth and event-date formats were standardized before age and month were derived, event dates recorded in different source formats were converted to standardized 2024 calendar dates. Sex labels and event-level categorical variables were harmonized across source files. Mucous stool weight was converted to a numerical variable when a valid quantitative value was available. Mucous stool weights were analyzed as recorded in the original routine records. Because weighing-device precision and rounding practices were not consistently documented, the measurement granularity could not be retrospectively standardized or verified. Missing or non-quantifiable observations were retained as missing rather than imputed. A detailed summary of data completeness for individual- and event-level variables is provided in Supplementary Table S1. Events were assigned to the month according to the standardized excretion date.

Physical phenotype was harmonized from the original routine records into three predefined categories: jelly-like, lard-like, and mixed jelly/lard. Because the retrospective source records did not contain duplicate independent ratings of the same event, formal inter-rater agreement statistics were not available. Therefore, potential observer-related variation in phenotype classification could not be quantified. Color categories were harmonized using a predefined mapping rule based on the original recorded color descriptions. Synonymous or closely equivalent descriptions were recoded into six standardized categories: brown, chartreuse, milky white, pale yellow, pink, and transparent. Records without a clear or interpretable color description were retained as not recorded and were not imputed. Because color was documented for only 188 of 674 events, it was used for descriptive purposes only and was not included in inferential analyses.

### 2.3. Ethical Considerations

This study was based exclusively on the retrospective analysis of pre-existing routine husbandry and health records collected during standard animal management. No experimental interventions, additional animal handling, biological sampling, or other procedures were performed on giant pandas specifically for the purposes of this study. Therefore, no prospective animal ethics approval was required for this retrospective record-based analysis. All data were analyzed in a de-identified manner.

### 2.4. Statistics

All statistical analyses were two-sided, with statistical significance defined as *p* < 0.05. Continuous variables were summarized using means and standard deviations (SDs), with medians and interquartile ranges (IQRs) additionally reported for skewed event-level variables; categorical variables were summarized as frequencies and percentages. Missing data were handled using a model-specific complete-case approach, and no missing values were imputed. For each analysis, only observations with non-missing data for all variables required by the corresponding model were included. Age was available for 189 of 190 pandas, and the individual with missing age was excluded only from analyses requiring age. Sex was available for 189 pandas, and the individual with missing sex was excluded from sex-related analyses. Body weight was available for 161 pandas; therefore, the exploratory univariable body-weight analysis was restricted to these individuals. The corresponding multivariable model included only pandas with complete data for body weight, age, and sex. At the event level, month was available for 673 of 674 events, mucous stool weight for 670 events, and physical phenotype for 672 events; analyses involving these variables were restricted to observations with the required data. Color information was substantially incomplete and was therefore retained for descriptive purposes only. Because this retrospective study analyzed an existing dataset, no a priori sample-size or power calculation was performed; statistical precision was assessed primarily using 95% confidence intervals. Statistical analyses were performed using Python version 3.13.5 with statsmodels version 0.14.6, NumPy version 2.3.5, and SciPy version 1.17.0.

At the individual level, occurrence of mucous stool excretion during the observation period was defined as a binary outcome (at least one event vs. no event). Logistic regression was used to evaluate associations of age and sex with event occurrence, with results expressed as odds ratios (ORs) and 95% confidence intervals (CIs). Annual event counts showed substantial overdispersion under a Poisson model (Pearson χ^2^ = 1126.98, df = 186, dispersion ratio = 6.06). Therefore, negative binomial regression was used to evaluate associations of age and sex with the number of mucous stool events per individual, with results expressed as incidence rate ratios (IRRs) and 95% CIs. Age was modeled as a continuous linear predictor in the primary models. Sensitivity analyses including a quadratic age term showed no evidence of non-linearity for event occurrence (*p* for age-squared = 0.996) or annual event frequency (*p* for age-squared = 0.129). Body weight was first evaluated in an exploratory univariable logistic regression and then in a multivariable model adjusted for age and sex among pandas with complete body-weight data (*n* = 161).

Monthly variation was analyzed using generalized estimating equations (GEEs) to account for repeated observations within the same panda. GEE was selected because the primary inferential interest was in marginal, population-averaged associations across the captive panda population rather than subject-specific effects conditional on individual random effects. Panda identity was specified as the clustering variable, and an exchangeable working correlation structure with robust sandwich standard errors was used for the primary analyses. Individual panda identity was the sole clustering variable in the GEE models. Study site was not treated as an additional clustering level because pandas were not permanently nested within a single site and could be transferred among CCRCGP bases during the observation period. Complete time-resolved residence histories were not consistently available; therefore, site was not included as a fixed covariate in the present analyses.

Monthly event counts were analyzed using a Poisson GEE model, with the logarithm of the number of calendar days in each month included as an offset. Because the number of pandas under observation remained constant throughout the study year, differences in monthly exposure were attributable only to month length. A binomial GEE model was used to evaluate monthly differences in the probability that an individual panda experienced at least one mucous stool event. Month was treated as a categorical variable. The overall effect of month was evaluated first. Month-versus-January comparisons were not prespecified and were conducted as exploratory post hoc contrasts, *p* values for the 11 comparisons were adjusted using the Holm method. Model convergence was verified for all fitted GEE models. Because GEE is a quasi-likelihood, population-averaged approach, conventional likelihood-based goodness-of-fit statistics are not directly applicable in the same manner as for likelihood-based mixed-effects models. Model robustness was therefore assessed by repeating the primary GEE analyses using an independence working correlation structure, which yielded materially similar conclusions.

Mucous stool weight was right-skewed and was therefore natural-log-transformed for inferential analyses. Monthly differences in mucous stool weight were evaluated using a Gaussian GEE model with month as a categorical predictor and the same clustering, working-correlation, and robust variance specifications described above. A Friedman test was not used because the data did not constitute a balanced matched repeated-measures design: individual pandas frequently had no mucous stool event in a given month and could contribute multiple events in other months. Phenotype-related differences in mucous stool weight were evaluated using an analogous Gaussian GEE model, with jelly-like stools as the reference category. Exponentiated model coefficients were reported as weight ratios with 95% confidence intervals (CIs). Descriptive distributions of mucous stool weight are presented using medians, interquartile ranges (IQRs), ranges, and box plots.

## 3. Results

### 3.1. Study Population and Mucous Stool Characteristics

The final study population comprised 190 giant pandas. Age was available for 189 animals and averaged 13.7 ± 6.1 years; body weight was available for 161 animals and averaged 117.0 ± 13.0 kg. Sex was recorded for 189 pandas (103 females and 86 males), with one individual missing sex information. During the observation period, 132 pandas (69.5%, 95% CI, 62.6–75.6%) experienced at least one mucous stool event, whereas 58 had no recorded event. A total of 674 mucous stool events were identified, including 374 events in females and 300 in males. The mean number of events per panda was 3.5 ± 4.7.

Mucous stool weight was available for 670 events and averaged 69.1 ± 55.1 g. Physical phenotype was documented for 672 events: 330 (49.1%) were jelly-like, 289 (43.0%) were lard-like, and 53 (7.9%) were mixed jelly/lard. Color was documented for 188 events; the most frequently recorded categories were milky white (64 events), pale yellow (63 events), and pink (44 events), while 486 events (72.1%) had no color record. Roundworm-positive findings were explicitly documented in 179 events (26.6%); the remaining 495 events had no positive record. The recorded deworming interval was available for 331 events and averaged approximately 47 ± 34 days (Table 1).

### 3.2. Individual Factors Associated with Mucous Stool Excretion

Age was not associated with whether a panda experienced at least one mucous stool event (OR per year = 0.988, 95% CI, 0.939–1.040, *p* = 0.652), and males did not differ significantly from females (OR = 0.800, 95% CI, 0.428–1.497, *p* = 0.485). Similarly, neither age nor sex was associated with the annual number of mucous stool events in negative binomial regression (age: IRR = 1.010, 95% CI, 0.977–1.044, *p* = 0.553, male vs. female: IRR = 0.979, 95% CI, 0.670–1.433, *p* = 0.915).

In an exploratory analysis restricted to the 161 pandas with available body-weight data, higher body weight was associated with lower odds of experiencing at least one mucous stool event (OR per kg = 0.961, 95% CI, 0.930–0.992, *p* = 0.015). This inverse association persisted after adjustment for age and sex in a multivariable logistic regression model (adjusted OR per kg = 0.961, 95% CI, 0.930–0.992, *p* = 0.015). In the same model, neither age (adjusted OR = 0.968, 95% CI, 0.912–1.028, *p* = 0.295) nor sex (male vs. female: adjusted OR = 1.256, 95% CI, 0.590–2.676, *p* = 0.554) was significantly associated with event occurrence. Because body-weight data were incomplete and measurements were not obtained at standardized time points relative to mucous stool events, this association was considered exploratory and should not be interpreted as evidence of a protective effect of higher body weight (Table 2).

### 3.3. Distribution and Concentration of Recurrent Mucous Stool Events Among Individuals

Annual mucous stool event counts were unevenly distributed across individuals. Among the 190 pandas, 58 (30.5%) had no recorded mucous stool event during 2024, whereas 132 (69.5%) experienced at least one event. The distribution of annual event counts was strongly right-skewed, with a small subset of pandas experiencing repeated episodes. The 19 pandas with the highest annual event frequencies, representing 10% of the study population, accounted for 260 of the 674 recorded events (38.6%). The most frequently affected panda experienced 40 events during the study year.

The contribution of these recurrent individuals also varied across months. In July, when the largest number of events was observed, the top 19 recurrent pandas accounted for 38 of 82 events (46.3%), whereas the remaining 171 pandas accounted for 44 events (53.7%). Thus, recurrently affected individuals contributed disproportionately to the overall event burden and made a substantial contribution to the elevated July count, although the July increase was not attributable exclusively to this subgroup (Figure S1).

### 3.4. Monthly Distribution of Mucous Stool Events

Among the 673 events with an interpretable month, the monthly event counts were 54, 39, 46, 69, 66, 71, 82, 50, 50, 40, 60, and 46 from January through December, respectively. July had the largest number of events (*n* = 82), followed by June (*n* = 71), April (*n* = 69), and May (*n* = 66). In the Poisson GEE model, month had a significant overall effect on event frequency (Wald chi-square = 30.22, df = 11, *p* = 0.0015). The unadjusted July-versus-January contrast yielded an RR of 1.519 (95% CI, 1.073–2.149, unadjusted *p* = 0.018); however, this comparison was not statistically significant after Holm correction for the 11 post hoc month-versus-January comparisons (adjusted *p* = 0.202).

The number of pandas experiencing at least one event also reached its maximum in July (*n* = 47), but the monthly probability that an individual panda experienced at least one event did not differ significantly overall (binomial GEE, *p* = 0.295). Thus, the monthly increase in total event frequency appears to reflect recurrent episodes among affected individuals rather than a substantial month-to-month expansion in the number of pandas affected (Figure 3).

### 3.5. Monthly Variation in Mucous Stool Weight

Mucous stool weight varied substantially among individual events but did not show a statistically significant monthly difference. Monthly mean weights ranged from 56.9 g in July to 76.9 g in April, and monthly medians were generally 50–60 g, with a median of 70 g in March. The Kruskal–Wallis test showed no significant difference in weight across months (H = 12.98, *p* = 0.295, Figure 4). These results indicate that the month-to-month variation observed in event frequency was not accompanied by a corresponding change in the amount of mucous material excreted per event.

### 3.6. Association Between Physical Phenotype and Mucous Stool Weight

Mucous stool weight differed significantly among the three physical phenotypes. Because mucous stool weight was natural-log-transformed for the Gaussian GEE analysis, model coefficients were exponentiated and are reported here as weight ratios. Lard-like stools were 1.318 times heavier than jelly-like stools (weight ratio = 1.318, 95% CI, 1.162–1.495; *p* < 0.0001), while mixed jelly/lard stools were 2.133 times heavier than jelly-like stools (weight ratio = 2.133, 95% CI, 1.733–2.626; *p* < 0.0001) and 1.619 times heavier than lard-like stools (weight ratio = 1.619, 95% CI, 1.340–1.955; *p* < 0.0001) (Figure 5).

## 4. Discussion

This multicenter retrospective study provides a large-scale characterization of mucous stool excretion in captive giant pandas and highlights several features that are relevant to both biological interpretation and routine husbandry management. Four principal findings emerged. First, mucous stool event frequency varied significantly across months during 2024, with the largest observed event count occurring in July, whereas the monthly probability that an individual panda experienced at least one event did not differ significantly. Second, recurrently affected individuals contributed disproportionately to the overall event burden but did not fully account for the higher event count observed in July. Third, mucous stool weight did not vary significantly across months, whereas visible physical phenotype was strongly associated with the amount of mucous material excreted. Finally, age and sex were not associated with mucous stool occurrence or annual event frequency, while an inverse association with body weight was identified only in an exploratory analysis and should therefore be interpreted cautiously. These findings suggest that variation in mucous stool excretion is characterized more by differences in event recurrence and event phenotype than by large changes in the proportion of pandas affected over time.

The within-year temporal pattern observed in the present study differs from that reported in the 15-year prospective study by Li et al. [4], in which mean mucous stool frequency was highest in October and the strongest monthly time-series effect was observed in August. In the present dataset, event counts increased from April through July, with July having the largest observed number of events. These differences may reflect variation in study population, institutional management, diet, bamboo availability, climate, or other environmental conditions [8,9]. Previous studies have linked bamboo intake and dietary transitions with fecal consistency and mucous stool occurrence, while broader changes in diet, habitat, and lifestyle have also been associated with alterations in the giant panda gut microbiome [3,10]. However, quantitative information on bamboo intake, bamboo species and plant parts consumed, temperature, humidity, and microbiome composition was not consistently available in the present retrospective dataset. Dietary and environmental conditions that vary across the year should therefore be regarded as plausible contributors rather than established causes of the observed monthly pattern. Importantly, because the study covered only a single calendar year, the higher event count observed in July 2024 cannot be interpreted as evidence of a reproducible seasonal pattern and requires confirmation using multi-year longitudinal data.

A particularly informative finding was the distinction between monthly event burden and the number of pandas affected. Although overall event frequency varied significantly across months, the monthly probability that an individual panda experienced at least one mucous stool event did not. The additional recurrence analysis provides further context for this dissociation. The 19 most frequently affected pandas, representing 10% of the study population, accounted for 38.6% of all recorded events and 46.3% of the events observed in July. Nevertheless, the remaining pandas still contributed 53.7% of July events, indicating that the increased July event count was not driven exclusively by a small group of highly recurrent individuals. Thus, the observed pattern is consistent with contributions from both persistent individual susceptibility and broader time-varying population-level influences. From a husbandry perspective, these findings support longitudinal monitoring of pandas with repeated episodes and systematic recording of previous-event history rather than relying solely on population-level monthly event counts. Future multi-year longitudinal analyses could incorporate individual-level random effects or frailty terms, together with repeated measurements of dietary and environmental exposures, to distinguish persistent between-panda susceptibility from broader population-level temporal effects.

In contrast to event frequency, the amount of mucous material excreted per event did not differ significantly across months. The repeated-measures Gaussian generalized estimating equation analysis of natural-log-transformed mucous stool weight showed no significant overall month effect. This suggests that factors contributing to the occurrence or recurrence of mucous stool episodes may not necessarily determine the amount of material excreted during an individual event. The observation that months with more events did not consistently have heavier mucous stools further supports this distinction. Prospective studies that record appetite, fecal output, bamboo intake, duration of gastrointestinal signs, and other clinical characteristics around each event would be valuable for determining whether event initiation, recurrence, and the amount of mucous material excreted are governed by different biological processes.

Visible physical phenotype, however, was strongly associated with mucous stool weight. After accounting for repeated observations within individual pandas, lard-like stools were approximately 1.32 times heavier than jelly-like stools, while mixed jelly/lard stools were approximately 2.13 times heavier than jelly-like stools and 1.62 times heavier than lard-like stools. These differences may reflect variation in physical composition, hydration, mucus accumulation, or other unmeasured characteristics of the excreted material. Nevertheless, the present study did not measure water content, lipid content, cellular composition, inflammatory markers, or biochemical characteristics of the mucus. Therefore, the three visible phenotypes cannot currently be interpreted as representing distinct pathological processes. In particular, greater mucous stool weight should not be considered evidence of more severe gastrointestinal disturbance, because standardized measures of clinical severity were not available. The heavier weight of mixed jelly/lard stools should therefore be interpreted as a phenotypic difference rather than a severity marker.

Despite this uncertainty regarding biological mechanisms, standardized phenotype classification may have practical value for veterinary surveillance and husbandry management. Recording phenotype together with mucous stool weight, recurrence history, appetite, activity, fecal output, and other gastrointestinal signs could provide a more structured description of individual episodes and improve longitudinal comparisons within the same panda. Such records may help identify animals with recurrent or changing patterns that warrant closer observation. Future prospective studies combining standardized phenotype assessment with clinical, biochemical, microbiological, and histological measurements will be necessary to determine whether specific visible phenotypes correspond to distinct gastrointestinal processes or clinical outcomes.

The exploratory inverse association between body weight and mucous stool occurrence also warrants cautious consideration. The association remained statistically significant after adjustment for age and sex, suggesting that these two demographic factors alone did not explain the observed relationship. Body weight may reflect several aspects of physiological status, including nutritional condition, dietary intake, body condition, reproductive status, and general health. However, these potential explanations cannot be evaluated with the present data. Body-weight information was available for only 161 of 190 pandas, exact weighing dates could not be consistently reconstructed, and measurements were not obtained at standardized time points relative to either the 2024 observation period or individual mucous stool events. Body condition scores and reproductive status were also not consistently available. Consequently, residual confounding and measurement-related uncertainty remain substantial, and the observed association should not be interpreted as evidence that higher body weight protects against mucous stool excretion. Prospective studies incorporating standardized longitudinal body weight, body condition, dietary intake, reproductive status, and gastrointestinal health measures will be required to determine whether this association is reproducible and biologically meaningful.

Several limitations should be considered when interpreting these findings. First, the retrospective design limits causal inference and depends on the completeness and accuracy of routine management records. Second, although the four study sites operated under a common institutional husbandry framework, they were geographically distinct and may have differed in climate, altitude, bamboo availability, weighing practice, phenotype classification, and event-recording procedures. In addition, pandas could be transferred among bases during the observation period, and complete time-resolved residence histories were not consistently available. Consequently, study site could not be incorporated as a stable individual-level covariate or additional clustering level, and residual site-level confounding cannot be excluded. Third, phenotype classification was derived from routine records generated by different staff, and duplicate independent assessments of the same events were unavailable; therefore, formal inter-rater agreement could not be evaluated, and observer-related misclassification remains possible. Fourth, the precision of mucous stool weight measurements and any rounding practices were not consistently documented, which may have reduced sensitivity to detect relatively small differences in weight. Fifth, body-weight and color information were incomplete to varying degrees, potentially introducing selection or recording bias. Sixth, quantitative information on actual bamboo intake, bamboo species and plant parts, environmental conditions, microbiome composition, behavior, and gastrointestinal clinical signs was not consistently available. Seventh, the event-centered dataset was not a complete daily follow-up dataset, and differences in observation intensity among individuals may therefore have influenced event ascertainment. Finally, the study covered only a single calendar year, so the observed within-year temporal pattern requires confirmation across multiple years.

Despite these limitations, the present study demonstrates the value of combining individual-level and event-level information while accounting for repeated observations within pandas. The results suggest that recurrently affected individuals deserve particular attention in longitudinal veterinary monitoring and that standardized documentation of mucous stool phenotype and weight may provide useful information beyond simple event counts. Future surveillance would benefit from prospectively recording event date, phenotype, weight, appetite and activity changes, fecal output, quantitative bamboo intake, environmental conditions, clinical findings, and time-resolved study-site information. Multi-year prospective datasets incorporating these measures would allow more rigorous evaluation of individual susceptibility, temporal variation, site effects, and potential biological mechanisms underlying mucous stool excretion in captive giant pandas.

## 5. Conclusions

This multicenter retrospective study characterized mucous stool excretion in 190 captive giant pandas during 2024. Mucous stool event frequency varied across months, with the largest number of events observed in July, whereas the proportion of pandas affected and the amount of mucous material excreted per event did not differ significantly across months. Recurrently affected individuals contributed disproportionately to the total event burden, and mucous stool weight differed markedly among physical phenotypes, with mixed jelly/lard stools being the heaviest. These findings highlight the importance of longitudinal monitoring of recurrently affected pandas and standardized recording of mucous stool phenotype and weight. Multi-year prospective studies incorporating dietary, environmental, clinical, and site-specific information are needed to confirm temporal patterns and clarify the underlying mechanisms.

## Figures and Tables

**Figure 2:**
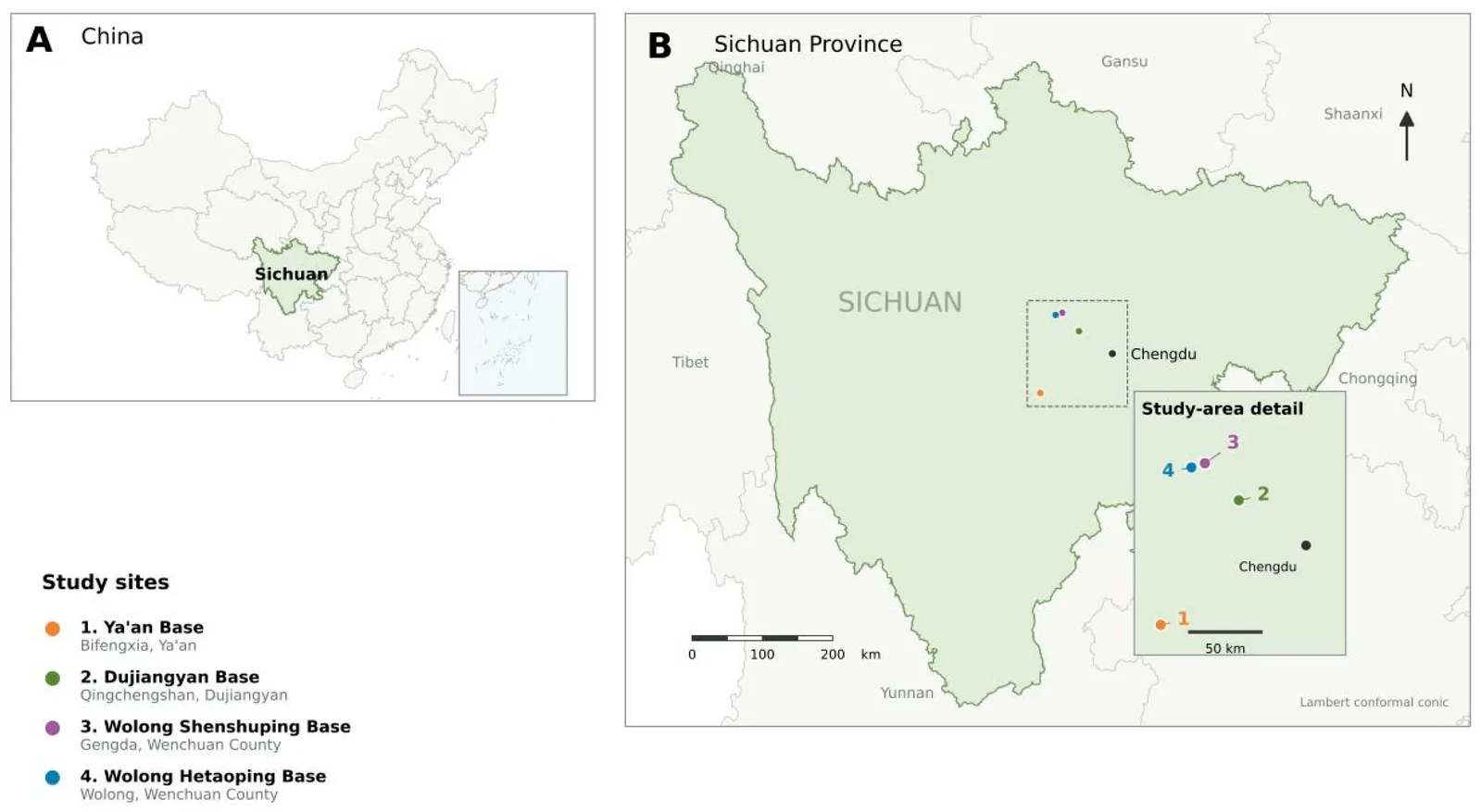
Geographic distribution of the four CCRCGP study sites in Sichuan Province, China. (**A**) Location of Sichuan Province within China. (**B**) Locations of the four study sites included in this retrospective multicenter study: Ya’an Base, Dujiangyan Base, Wolong Shenshuping Base, and Wolong Hetaoping Base of CCRCGP.

**Figure 3:**
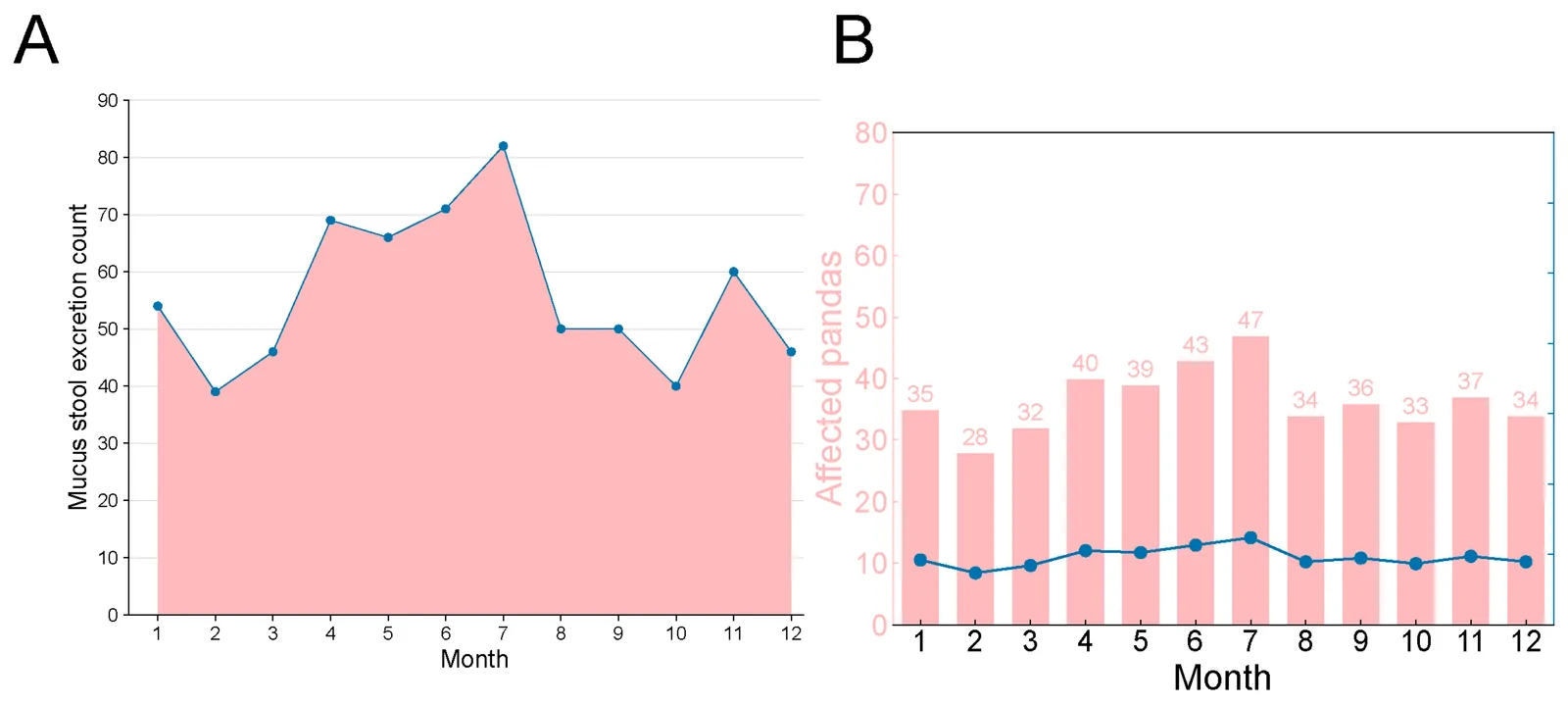
Monthly distribution of mucous stool excretion in captive giant pandas during 2024. (**A**) Monthly number of mucous stool excretion events. (**B**) Number of giant pandas experiencing at least one mucous stool event in each month, blue dots indicate the corresponding monthly percentage of affected pandas.

**Figure 4:**
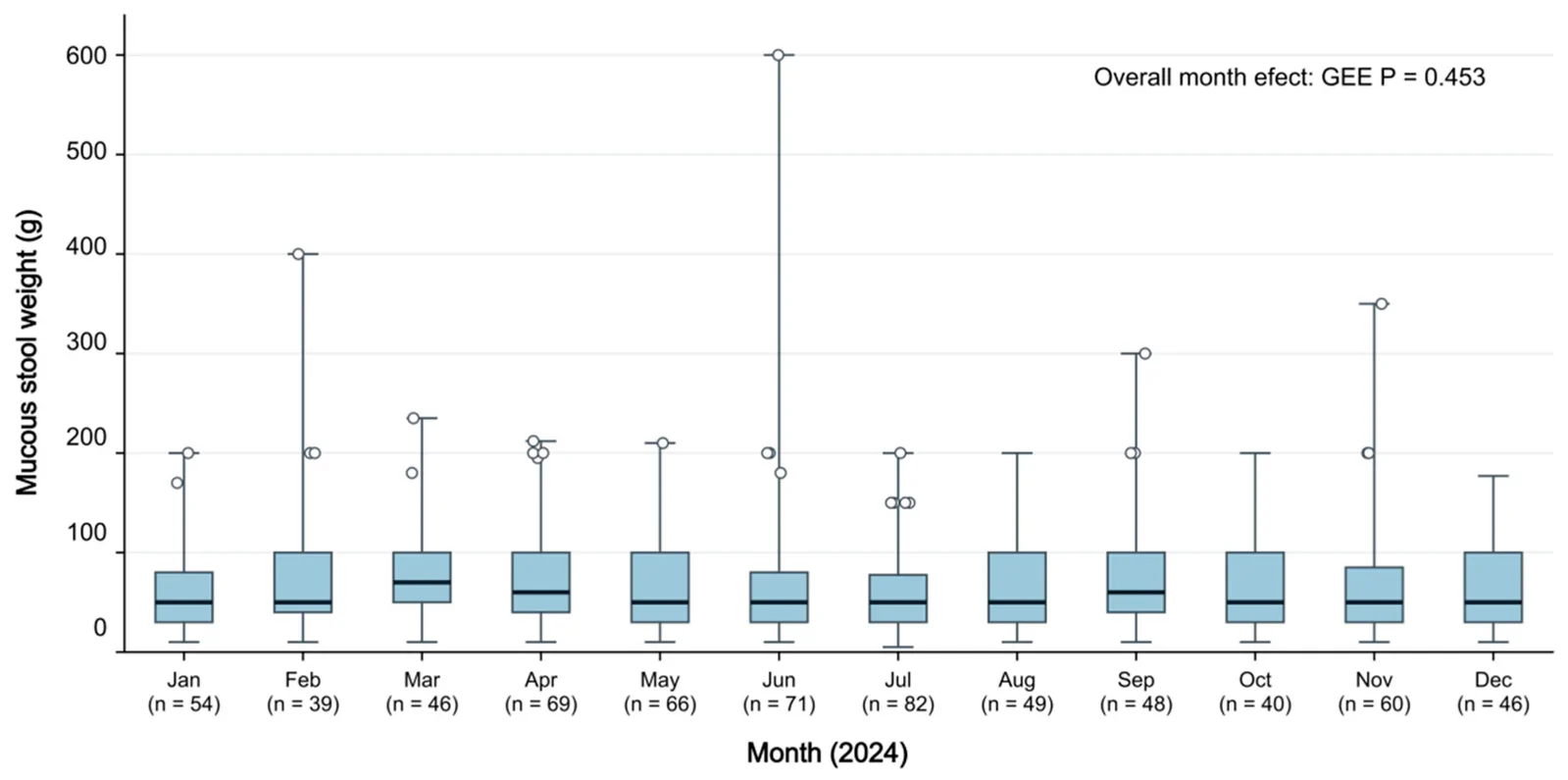
Monthly variation in mucous stool weight in captive giant pandas. Box plots show the distribution of mucous stool weight for each month. The center line represents the median, the box represents the interquartile range (IQR), and circles indicate individual observations beyond the whiskers. No significant overall difference was detected across months in the repeated-measures GEE analysis (Wald χ^2^ = 10.88, df = 11, *p* = 0.453).

**Figure 5:**
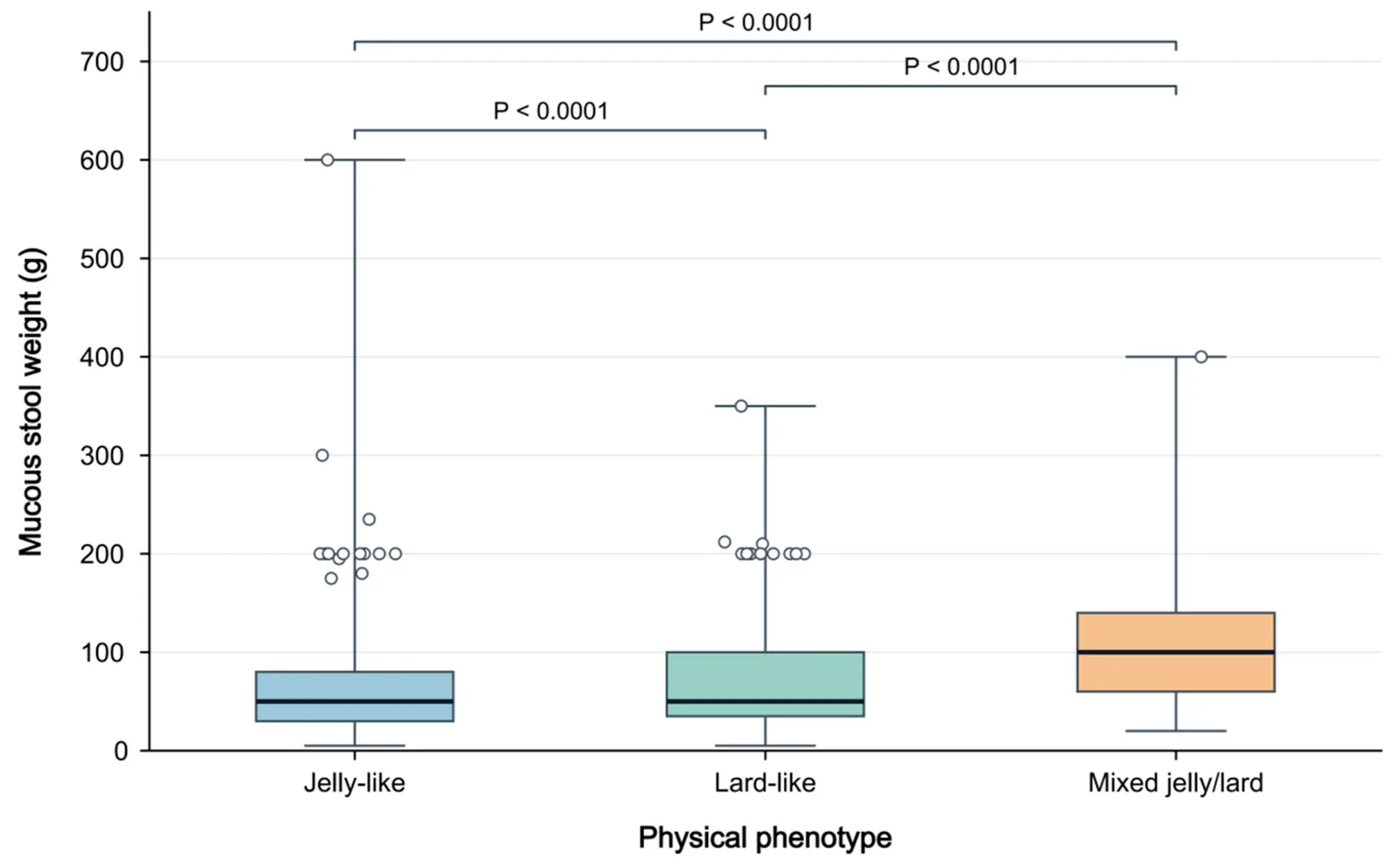
Mucous stool weight according to physical phenotype. Box plots summarize events with numerical weight and recorded phenotype: jelly-like (*n* = 327), lard-like (*n* = 289), and mixed jelly/lard (*n* = 53). Boxes indicate the interquartile range (25th–75th percentiles), horizontal lines indicate medians, and whiskers extend to the most extreme observations within 1.5 × IQR, and circles indicate individual observations beyond the whiskers. Observations beyond the whiskers are shown individually. Brackets indicate pairwise comparisons derived from the repeated-measures Gaussian GEE model of natural-log-transformed mucous stool weight, with panda identity as the clustering variable, an exchangeable working correlation structure, and robust standard errors. The comparisons shown are lard-like versus jelly-like, mixed jelly/lard versus jelly-like, and mixed jelly/lard versus lard-like; all three yielded two-sided *p* < 0.0001.

**Table 2:** Statistical analyses of mucous stool occurrence, event frequency, and weight.

Analysis	Effect Estimate	95% CI/Test Statistic	*p* Value	Interpretation
Occurrence of mucous stool excretion				
Age	OR = 0.988	95% CI 0.939–1.040	0.652	Not significant
Sex	OR = 0.800	95% CI 0.428–1.497	0.485	Not significant
Annual number of mucous stool events				
Age	IRR = 1.010	95% CI 0.977–1.044	0.553	Not significant
Sex	IRR = 0.979	95% CI 0.670–1.433	0.915	Not significant
Monthly variation in mucous stool events				
Month	-	Wald chi-square = 30.22, df = 11	0.0015	Significant overall month effect
July vs. January	RR = 1.519	95% CI 1.073–2.149	Holm-adjusted *p* = 0.202	Post hoc contrast not significant after correction
Monthly probability of at least one event				
Month	-	Wald chi-square = 12.97, df = 11	0.295	Not significant
Mucous stool weight				
Month	-	Wald chi-square = 10.88, df = 11	0.453	No significant monthly difference
Lard-like vs. Jelly-like	Weight ratio = 1.318	95% CI 1.162–1.495	<0.0001	Lard-like stools were heavier
Mixed vs. Jelly-like	Weight ratio = 2.133	95% CI 1.733–2.626	<0.0001	Mixed stools were heavier
Mixed vs. Lard-like	Weight ratio = 1.619	95% CI 1.340–1.955	<0.0001	Mixed stools were heavier
Exploratory multivariable analysis				
Body weight, per kg	Adjusted OR = 0.961	95% CI 0.930–0.992	0.015	Inverse association, interpret cautiously
Age	Adjusted OR = 0.968	95% CI 0.912–1.028	0.295	Not significant
Sex, male vs. female	Adjusted OR = 1.256	95% CI 0.590–2.676	0.554	Not significant

## Data Availability

The data presented in this study are available upon request from the corresponding author.

## Supplementary Materials

The following supporting information can be downloaded at: https://www.mdpi.com/article/10.3390/ani16193047/s1, Figure S1. Distribution and concentration of recurrent mucous stool events among captive giant pandas during 2024. (A) Distribution of the annual number of mucous stool events per panda among all 190 individuals. (B) Cumulative proportion of total mucous stool events contributed by pandas ranked according to annual event frequency. (C) Monthly distribution of mucous stool events according to recurrence status. Table S1. Data completeness of individual- and event-level variables.
