# Supplementary material for: A Retrospective Study on Mucous Stool Excretion in Giant Pandas: Statistical Analysis Based on CCRCGP Data
Source: Animals (Basel). 2026 Sep 28;16(19):3047. doi: 10.3390/ani16193047 (PMC13663466; doi:10.3390/ani16193047)
Supplement: Supplementary file 1 [file animals-16-03047-s001.zip › animals-4534694-supplementary.pdf]

Supplementary Materials

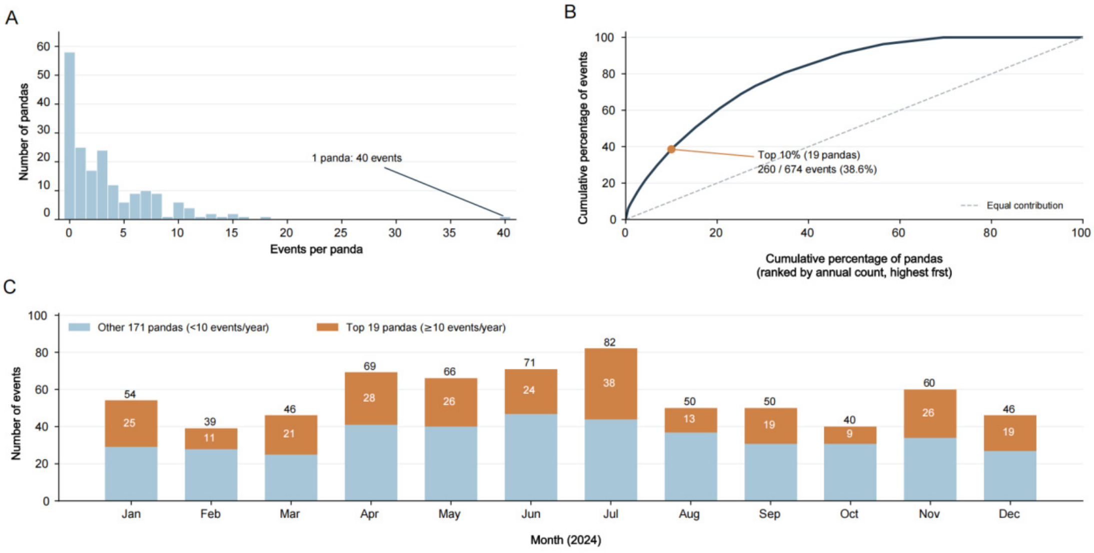

Figure S1. Distribution and concentration of recurrent mucous stool events among captive giant pandas during 2024. (A) Distribution of the annual number of mucous stool events per panda among all 190 individuals. (B) Cumulative proportion of total mucous stool events contributed by pandas ranked according to annual event frequency. (C) Monthly distribution of mucous stool events according to recurrence status.

Supplementary Table S1. Data completeness of individual- and event-level variables

Variable	Available	Missing / not recorded	Completeness
Age	189/190	1	99.5%
Sex	189/190	1	99.5%
Body weight	161/190	29	84.7%
Event month	673/674	1	99.9%
Mucous stool weight	670/674	4	99.4%
Physical phenotype	672/674	2	99.7%
Color	188/674	486	27.9%
